# Weight Loss Strategies Associated With Type 2 Diabetes Duration: A Population‐Based Optimal Intervention Window From the NHANES

**DOI:** 10.1155/jdr/8319702

**Published:** 2026-04-27

**Authors:** Maximilian Andreas Storz, Roman Huber, Jochen Seufert

**Affiliations:** ^1^ Department of Internal Medicine II, Centre for Complementary Medicine, Medical Center – University of Freiburg, Faculty of Medicine, University of Freiburg, Freiburg, Germany, uni-freiburg.de; ^2^ Department of Internal Medicine II, Division of Endocrinology and Diabetology, Medical Center – University of Freiburg, Faculty of Medicine, University of Freiburg, Freiburg, Germany, uni-freiburg.de

**Keywords:** diabetes, glycemic control, weight management

## Abstract

**Background:**

Obesity is the most important risk factor in the development and progression of type 2 diabetes. Its tight link to impaired glycemic control highlights the importance of effective weight loss actions (WLAs). Population‐based studies investigating associations between type 2 diabetes duration and the odds for undertaking WLAs are scarce. Some studies showed that individuals with incident type 2 diabetes reported at least minimal changes in physical activity and diet, whereas others suggested that lifestyle modifications are generally short‐lived and decrease with a longer diabetes duration.

**Methods:**

Using cross‐sectional data from the National Health and Nutrition Examination Surveys (NHANES), we hypothesized that the odds for undertaking self‐reported WLAs within the last 12 months would decrease with a longer diabetes duration. Multivariable logistic regression models with restricted cubic splines were used to estimate marginal predicted probabilities for undertaking WLAs in 2118 participants with type 2 diabetes. Frequently reported WLAs were analyzed.

**Results:**

Results suggested a nonlinear relationship between the time elapsed since diagnosis and the odds for taking WLAs. The marginal predicted probability for taking WLAs was highest in the first year after diagnosis, e.g., 0.57 (95% CI: 0.50–0.65). Probabilities fell thereafter and dropped to 0.46 after 7 years (95% CI: 0.42–0.50). The time window from three to 6 years after diagnosis was identified as a potentially important intervention window, as marginal predicted probabilities dropped by 0.02 (95% CI: −0.38 to −0.001) for each additional year after diagnosis (*p* = 0.042). Eating less to lose weight and exercising more were the most commonly reported WLAs, with 68.78% and 45.73%, respectively. Time elapsed since diagnosis was associated with the number of WLAs taken.

**Conclusion:**

Results suggest a significant decrease of WLAs from 3 to 6 years after the diabetes diagnosis, which population‐based weight loss strategies should take into account. Early WLAs are crucial before motivation decreases further.

## 1. Introduction

Type 2 diabetes represents a major lifestyle‐related public health problem and exerts a tremendous toll on healthcare systems globally [1, 2]. Data from the Global Burden of Disease study suggested that ~462 million individuals were affected by type 2 diabetes in 2017, corresponding to more than 6% of the world’s total population [3]. At present, more than 1 million deaths per year may be attributed to type 2 diabetes alone [3, 4].

Overweight and obesity are the most important risk factors in the development and progression of type 2 diabetes [5]. Numerous studies support the association between excess body weight and impaired glycemic control in affected individuals [6, 7]. Modest weight loss, on the other hand, may lead to substantial improvements in glycemic control outcomes [8].

The advent of GLP‐1 (Glucagon‐like Peptide‐1) receptor agonists represented a pharmacological milestone in the treatment of type 2 diabetes [9]. While beneficially affecting body weight and glycemic control, GLP‐1 receptor agonists also have well‐known side effects (including nausea, diarrhea, and vomiting), and their widespread use results in substantial healthcare costs [9]. A Lancet Diabetes & Endocrinology editorial emphasized that the view that “[…] GLP‐1 receptor agonists will be the solution to […]” public health emergencies, such as obesity and diabetes, “[…] risks undermining prevention efforts” and amplifies “[…] health inequalities given the elevated costs of these drugs.” Against this background, it is of utmost importance to encourage lasting lifestyle changes to promote a healthier bodyweight in individuals with type 2 diabetes [2].

Various studies used nationally representative data from the US‐based National Health and Nutrition Examination Surveys (NHANES) to investigate the most common weight loss strategies adopted by Americans *with* and *without* type 2 diabetes [10, 11]. Wang et al. [10] suggested that 86.1% of Americans with type 2 diabetes were overweight or obese. Almost 60% of individuals with overweight or obesity undertook weight loss actions (WLAs). While most of the adopted actions were diet‐related, a noticeable share of participants also reported potentially harmful strategies, such as smoking or taking laxatives/vomiting.

The aforementioned analysis by Wang et al. specifically focused on the type of weight loss action, whereas potential associations between the odds for undertaking WLAs and time trajectories (e.g., the time elapsed since type 2 diabetes diagnosis) were not explored. Insights into such associations would allow for more targeted population‐based public health strategies and prevention efforts and could potentially facilitate resource allocation. From a public health perspective, such data would be helpful to identify a time window when individual intrinsic motivation to modify lifestyle behaviors for weight loss starts to decline in individuals with type 2 diabetes and when external stimuli in the form of population‐based strategies that improve social, physical, and environmental contexts for weight loss could counteract this loss of motivation. Data on time trajectories and health behavior modifications in patients with type 2 diabetes, however, is generally scarce [12–14], and further research in this area is warranted.

The present study thus sought to explore said associations using data from the NHANES [15]. More specifically, we hypothesized that the odds for undertaking WLAs would decrease with a longer time elapsed since the type 2 diabetes diagnosis (primary hypothesis). Based on the extant literature, time was considered a crucial external factor why individuals with type 2 diabetes would lose motivation and capability in weight loss/maintenance efforts [16–18]. We also hypothesized that a longer diabetes duration would be associated with a lower number of WLAs taken. Finally, we conducted an exploratory analysis (secondary outcome) to identify the most frequent WLAs reported by NHANES participants with type 2 diabetes and investigated whether the predicted probabilities for certain (potentially harmful) actions increase with a longer time elapsed since diagnosis.

## 2. Materials and Methods

### 2.1. NHANES 2009–2018

The NHANES is a US‐based nationwide survey designed by the National Center for Health Statistics (NCHS) to assess population‐level estimates of the prevalence of health‐related outcomes among the noninstitutionalized civilian population [15, 19, 20]. The NHANES recruits ~5000 participants annually and is characterized by a complex multistage, stratified, clustered, and probability sampling design [15, 20]. NHANES data is frequently used in biomedical research, particularly when it comes to diabetes‐related questions at the population level [21]. The NHANES received ethical approval from the NCHS Ethics Review Board [15].

### 2.2. Primary Outcome

The primary outcome for this study was a self‐reported weight loss attempt in the past 12 months. Data was drawn from the NHANES weight history module [22], which included the following question: “During the past 12 months, {have you/has sample person} tried to lose weight?.” Potential replies included “yes” and “no”; participants who refused an answer were excluded.

### 2.3. Secondary Outcome

The secondary outcome was the total number of WLAs taken. For this, we considered all prespecified WLAs queried in the NHANES weight history module [22]. All variables were recoded in a binary manner (0 = action not taken; 1 = action taken), and the total sum of action was calculated. WLAs included: eating less to lose weight (WHD080A); switching to foods with lower calories (WHD080B); eating less fat to lose weight (WHD080C); exercising to lose weight (WHD080D); skipping meals (WHD080E); eating diet foods or products (WHD080F); using liquid diet formulas (WHD080G); joining a weight loss program (WHD080H); taking prescription diet pills (WHD080I); taking non‐RX supplements to lose weight (WHD080J); taking laxatives or vomiting (WHD080K); drinking a lot of water (WHD080M); following a special diet (WHD080N); eating fewer carbohydrate (WHD080O); starting smoking or beginning to smoke again (WHD080P); eating more fruits, vegetables, and salads (WHD080Q); changing eating habits (WHD080R); eating less sugar, candy, and sweets (WHD080S); eating less junk food or fast food (WHD080T); having weight loss surgery (WHD080U); and other nonfurther specified measures taken (WHD080L). These prespecified WLAs were mostly physical activity‐ and diet‐related and not necessarily complete.

### 2.4. Exposure

The exposure was self‐reported diabetes and, more specifically, the time elapsed since diabetes diagnosis (i.e., diabetes duration). This information was obtained from the NHANES diabetes questionnaire [23]. This questionnaire provides personal interview data with regard to diabetes duration, diabetes medication usage (insulin or oral hypoglycemic medications), and other diabetes‐related questions. Age at diabetes diagnosis was obtained from the questionnaire directly. The time elapsed since diagnosis was calculated as follows: age at participation *minus* age at diagnosis.

Only participants who had been previously told by a doctor that they had diabetes were considered (e.g., participants replying “yes” to the NHANES question diq010). So‐called borderline cases were not considered. Diabetes type classification followed the multistep treatment‐based algorithm by Mosslemi et al. [24] as described earlier in detail. Participants with type 1 diabetes were excluded (see below).

### 2.5. Covariables

The present study contains a large set of covariables known to be associated with diabetes and overweight/obesity. Sociodemographic factors were selected based on previous studies by Mokari‐Yamchi and Rosenkranz and Wang et al. [10, 11]. Supporting Information 1: Table S1 lists all covariables for this study. Anthropometric data at participation was drawn from the NHANES body measures module. This included BMI (in kg/m^2^), which was used as a continuous variable in the present study. However, we also considered current self‐reported body weight and self‐reported body weight 12 months prior to the interview. Various composite variables were built (e.g., weight alignment between measured and self‐reported weight) for additional insights into the examined cohort.

### 2.6. Inclusion and Exclusion Criteria

Only participants with a complete dataset were included; participants with missing data on any variable of interest were not considered. This study included only participants with type 2 diabetes. Participants with type 1 diabetes or “possible type 2 diabetes” as per the Mosslemi [24] classification were not considered. The study covered all continuous NHANES cycles from 2009 to 2018.

### 2.7. Statistical Analysis

Statistics were computed in Stata 19 (StataCorp. 2025. Stata Statistical Software: Release 19. College Station, TX: StataCorp LLC. The “. svyset” and “. svy” commands were used to account for the complex NHANES survey design characteristics and the required population weights [19, 20]. A 10‐year‐interview weight was constructed for this analysis. To preserve the survey design, we performed unconditional subclass analyses when comparing participants with type 2 diabetes *with* and *without* a weight loss intention within the last year. Weighted proportions with their corresponding 95% confidence interval (CI) were provided for all categorical variables. The reliability of all weighted proportions was assessed based on NCHS proportion standards as described earlier [20, 25]. Weighted scatter plots with frequency weights were used to visualize the data.

Subsequently, we constructed multivariable logistic regression models to predict the likelihood of a weight loss attempt in the last year depending on the time elapsed since diagnosis. The latter was entered as a continuous predictor, and — for explorative purposes only — as a categorical predictor based on quartiles. To account for a potential nonlinear relationship between both variables, we also employed regression models with restricted cubic splines with *n* = 5 knots. For this, we used the user‐written “f_able” Stata command by Rios‐Avilla [26]. The model building strategy, including candidate predictor selection, model definition and model refinement followed the proposed steps by Heeringa et al. [27]. Based on the obtained models, we visualized predicted probabilities depending on time elapsed since diagnosis and other covariables using Stata’s margins function [20]. Negative binomial regression was used to assess potential relationships between the number of WLAs undertaken and the time since diagnosis. Alpha was set to 0.05. This research followed the STROBE reporting guidelines for observational studies [28].

## 3. Results

**Figure 1:**
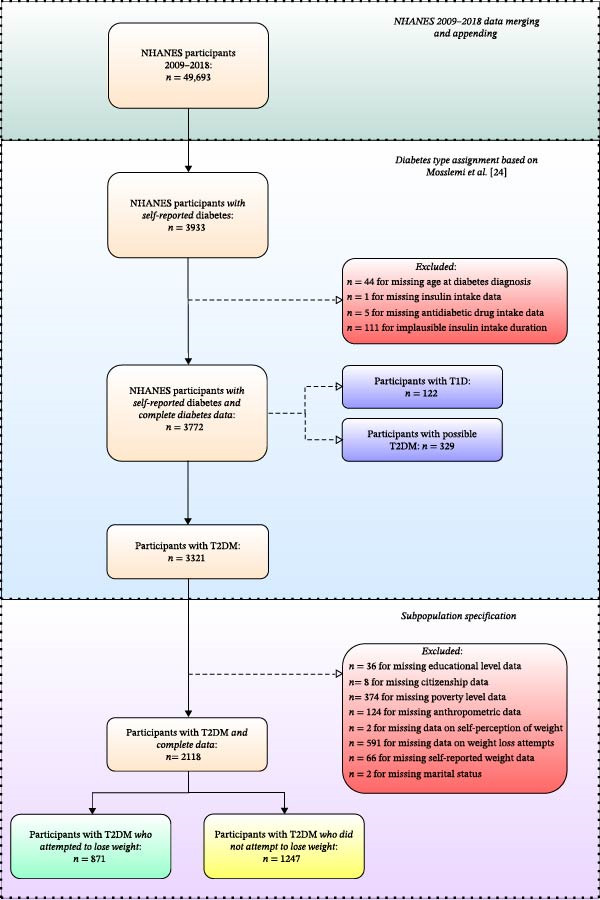
Participant inclusion flowchart: NHANES 2009–2018. Participants with missing data were excluded step‐by‐step. The final sample comprised *n* = 2118 participants with type 2 diabetes, thereof *n* = 871 who attempted to lose weight in the past year. T1*D* = Type‐1‐Diabetes. T2DM = Type 2 diabetes mellitus. *N* = 417 participants were added from the 2009/2010 cycle; *n* = 393 participants were added from the 2011/2012 cycle; *n* = 401 participants were added from the 2013/2014 cycle; *n* = 429 participants were added from the 2015/2016 cycle; and *n* = 478 participants were added from the 2017/2018 cycle.

The final sample analyzed in this study comprised *n* = 2118 participants with type 2 diabetes (Figure 1). This may be extrapolated to represent *n* = 12,825,867 US Americans. The weighted proportion of participants undertaking WLAs within the last year was 48.25%; 51.75% denied any weight loss attempts in this period.

Table 1 displays the sample characteristics depending on self‐reported weight loss intention. Participants with type 2 diabetes who attempted to lose weight tended to be younger, of non‐Hispanic White ethnicity, of higher educational level, and lived above the poverty threshold. More than 91% of participants attempting to lose weight considered themselves overweight (mean measured BMI: 35.51 kg/m^2^). While a large difference in mean BMI was observed between those with and without weight loss attempts in the past year (−4.96 kg/m^2^), both groups did neither differ with regard to the time elapsed since diagnosis nor with regard to the age at diagnosis. Supporting Information 1: Figure S1 provides additional insights into the distribution of both key variables in participants with type 2 diabetes attempting to lose weight. Participants with type 2 diabetes who intended to lose weight undertook, on average, *n* = 4.44 WLAs within the last 12 months (Supporting Information 2: Table S2).

**Table 1:** Sample characteristics of *n* = 2118 NHANES participants with type 2 diabetes by self‐reported weight loss intentions in the past year (NHANES 2009–2018).

Variable	Weight loss attempt past year: yes (*n* = 871)	Weight loss attempt past year: no (*n* = 1247)	*p*‐Value
Sex			*p* = 0.108 ^∗^
Male	49.33% (44.61–54.06)	54.54% (50.38–58.64)	—
Female	50.68% (45.94–55.39)	45.46% (41.36–49.62)	—
Ethnicity/race			***p* = 0.019** ^∗^
Mexican American	9.12% (6.62–12.43)	10.68% (7.98–14.14)	—
Other Hispanic	4.62% (3.40–6.27)	6.23% (4.77–8.09)	—
Non‐Hispanic White	65.04% (59.73–69.96)	56.25% (51.39–61.00)	—
Non‐Hispanic Black	12.03% (9.75–14.74)	14.27% (11.62–17.39)	—
Other Race	9.19% (6.90–12.14)	12.58% (10.14–15.50)	—
Age (years)	59.11 (58.00–60.21)	63.39 (62.44–64.35)	***p* < 0.001** ^∗∗^
Education level			***p* < 0.001** ^∗^
Less than 9th grade	6.19% (4.60–8.28)	13.26% (11.16–15.67)^‡^	—
9–11th grade	11.26% (8.72–14.41)	15.37% (12.66–18.55)^‡^	—
High school graduate/GED	23.77% (20.05–27.95)	25.71% (21.73–30.13)	—
Some college or AA degree	33.54% (28.91–38.52)	29.40% (25.01–34.31)	—
College graduate or above	25.24% (20.55–30.59)	16.26% (13.16–19.93)^‡^	—
Marital status			*p* = 0.060 ^∗^
Living with a partner/married	66.24% (61.51–70.66)	63.70% (60.95–66.36)	—
Divorced/separated/widowed/	24.67% (20.88–28.89)	29.89% (27.24–32.69)^‡^	—
Never married	9.09% (6.56–12.47)	6.41% (4.96–8.24)	—
Citizenship status			***p* = 0.001** ^∗^
US citizen by birth or naturalization	94.33% (92.25–95.87)	90.41% (88.46–92.07)^‡^	—
Not a citizen of the US	5.67% (4.13–7.75)	9.59% (7.93–11.54)^‡^	—
Ratio of family income to poverty			***p* = 0.002** ^∗^
<1 (below poverty threshold)	13.59% (11.19–16.42)	19.34% (16.36–22.72)^‡^	—
≥1 (above poverty threshold)	86.41 (83.58–88.81)	80.66% (77.28–83.64)^‡^	—
Number of people in the household	2.63 (2.51–2.75)	2.63 (2.51–2.75)	*p* = 0.973 ^∗∗^
Time elapsed since diagnosis	9.89 (8.88–10.90)	10.60 (9.97–11.24)	*p* = 0.183 ^∗∗^
Age when received diabetes diagnosis	49.21 (47.94–50.49)	52.79 (51.74–53.84)	*p* = 0.183 ^∗∗^
Body mass index (measured) in kg/m^2^	35.51 (34.70–36.33)	30.55 (29.90–31.20)	***p* < 0.001** ^∗∗^
Self‐assessed body weight			***p* < 0.001** ^∗^
Overweight	91.38% (88.19–93.77)	53.77% (49.56–57.92)^‡^	—
Underweight	0.51% (0.21–1.20)^†^	4.45% (3.35–6.01)^‡^	—
About the right weight	8.11% (5.82–11.20)	41.74% (37.83–45.75)^‡^	—
Self‐reported *minus* measured weight (kg)	−0.89 (−1.25−(−0.49))	0.02 (−0.44–0.48)	***p* = 0.004** ^∗∗^
Self‐reported weight change past year (kg)	1.91 (1.00–2.82)	−0.83 (−1.34–0.32)	***p* < 0.001** ^∗∗^
Measured weight change past year (kg)	2.78 (1.75–3.81)	−0.85 (−1.63–0.07)	***p* < 0.001** ^∗∗^

*Note:* Table 1 shows weighted proportions. Means with their 95% confidence interval are provided for continuous, normally distributed variables. Categorical variables are shown as weighted proportions (95% confidence interval). All weighted proportions can be considered reliable, as per the recommendations of Parker et al. [25], except for those marked with a “^†^” symbol, which denotes an unreliable proportion. The “^‡^” symbol denotes significant between‐group differences in the weighted proportions.

^∗^ *p*‐Value based on Stata’s design‐adjusted Rao–Scott test.

^∗∗^ *p*‐Value based on a regression analysis followed by adjusted Wald tests.

Figure 2 displays a series of weighted scatterplots showing associations between the time elapsed since diabetes diagnosis, the number of WLAs taken, and the participant’s body weight. A weak inverse correlation between the time elapsed since diagnosis and the number of actions taken (*r* = −0.11, *p* = 0.011) was found.

Figure 2Scatter plots displaying associations between the time elapsed since diabetes diagnosis, the number of weight loss actions taken, and the participant’s body weight. All plots are based on *n* = 871 participants with type 2 diabetes who attempted to lose weight in the past year. (A) shows a weak inverse correlation between the time elapsed since diagnosis (in years) and the number of actions taken (*r* = −0.11, *p* = 0.011). No association was found between the time elapsed since diagnosis and measured weight change within the last year (B) and self‐reported weight change within the last year (C). (D) suggests no correlation between the time elapsed since diagnosis and weight alignment (measured vs. reported weight during the examination).

**Figure:**
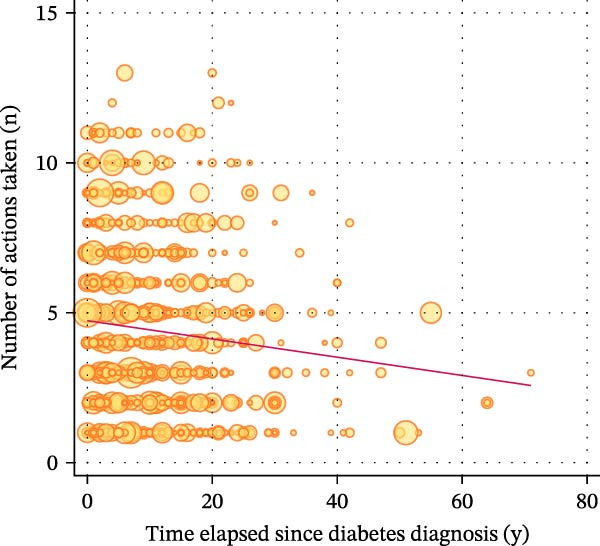
(A)

**Figure:**
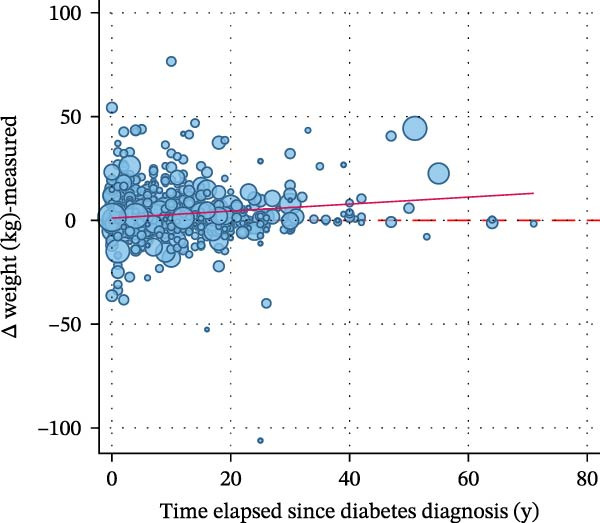
(B)

**Figure:**
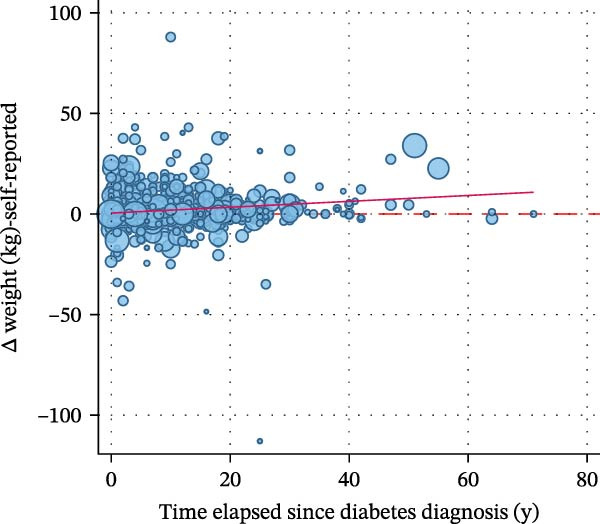
(C)

**Figure:**
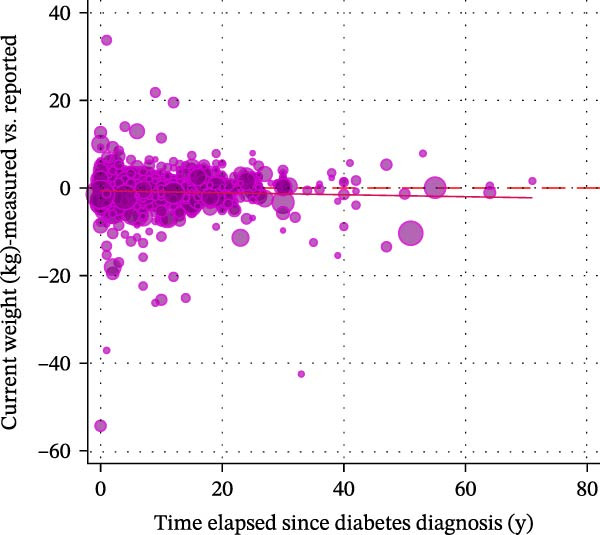
(D)

**Figure 3:**
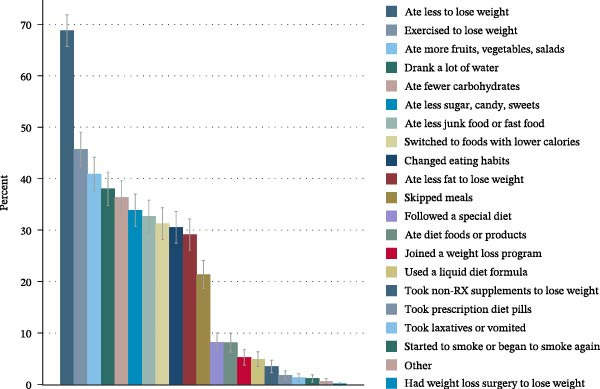
Bar chart displaying the most commonly reported weight loss strategies among *n* = 871 participants with type 2 diabetes undertaking weight loss actions in the NHANES (2009–2018). Eating less to lose weight and exercising were the most commonly reported weight loss strategies, with 68.78% and 45.73%, respectively. Harmful practices, such as taking laxatives/vomiting or smoking, were reported by 1.31% and 1.17%, respectively. Eating more fruits, vegetables, and salads, as well as drinking a lot of water, ranked second and third among the most frequently reported practices (40.91% and 38.04%, respectively).

Weight alignment (measured vs. self‐reported weight) was significantly more accurate in those who denied weight loss attempts (Table 1). Nevertheless, no correlation between the time elapsed since diagnosis and weight alignment was found (Figure 2, panel D). Figure 3 shows the most frequently reported weight loss strategies among the examined participants with type 2 diabetes. Eating less to lose weight and exercising were the most commonly reported weight loss strategies, with 68.78% and 45.73%, respectively. Potentially harmful practices, such as taking laxatives/vomiting or smoking, were reported by 1.31% and 1.17%, respectively. Eating more fruits, vegetables, and salads, as well as drinking a lot of water, ranked second and third among the most frequently reported dietary practices (40.91% and 38.04%, respectively). Supporting Information 1: Figure S3 displays weight loss trajectories in the examined sample.

We subsequently investigated whether the time elapsed since diabetes diagnosis was associated with an attempt to lose weight within the last 12 months (Supporting Information 1: Figure S4). A logistic regression model with time elapsed since diagnosis as a continuous predictor and with race/ethnicity, sex, poverty status, educational level, and marital status as covariates was built. Time elapsed since diabetes diagnosis was not associated with the likelihood of undertaking a weight loss action within the last year (OR: 0.99, CI: 0.98–1.01, *p* = 0.357). When entering time elapsed since diagnosis as a quartile‐based categorical variable, a significant association between both variables was found (not shown). This suggested a nonlinear relationship. We thus reexamined the association based on a logistic regression model with restricted cubic splines with *n* = 5 knots (Figure 4). Said model supported the aforementioned assumption. Estimated marginal predicted probabilities for undertaking a weight loss attempt in the last year were highest in the first months (0 years elapsed) after diagnosis (0.57; 95% CI: 0.50–0.65) and fell thereafter (panel A). Marginal predicted probabilities were lowest 12 years after a diabetes diagnosis (0.46; 95% CI: 0.41–0.51). After this nadir, a gradual increase in the predicted probabilities was observed. Predicted probabilities differed substantially depending on the time elapsed since diagnosis; e.g., predicted probabilities differed significantly when comparing 0 years (first months) after diagnosis vs. 5 years after diagnosis (*p* = 0.044). The time frame from 3 years to 6 years after diagnosis was identified as a potentially crucial time window, as marginal predicted probabilities dropped by 0.02 (95% CI: −0.38 to −0.001) for *each* additional year after diagnosis within that time window (*p* = 0.042).

Figure 4Associations between the time elapsed since diabetes diagnosis and the predicted probability of undertaking weight loss actions based on a logistic regression model with restricted cubic splines with *n* = 5 knots in *n* = 2118 observations with type 2 diabetes. Estimated marginal predicted probabilities for undertaking a weight loss attempt in the last year were highest in the first months (0 years elapsed) after diagnosis (0.57; 95% CI: 0.50–0.65) and fell thereafter (A). Marginal predicted probabilities dropped to 0.46 after 7 years (0.46; 95% CI: 0.42–0.50) and remained constant at this level. After 12 years, a gradual increase in predicted probabilities was observed. Predicted probabilities differed substantially depending on the time elapsed since diagnosis; e.g., predicted probabilities differed significantly when comparing 0 years (first months) after diagnosis vs. 5 years after diagnosis (*p* = 0.044). The time frame from 3 years to 6 years after diagnosis was identified as the crucial time window, as marginal predicted probabilities dropped by 0.02 (95% CI: −0.38 to −0.001) for each additional year after diagnosis (*p* = 0.042). (B–D) These depict marginal predicted probabilities by sex, educational level, and race/ethnicity, respectively. HSG = High School Graduate.

**Figure:**
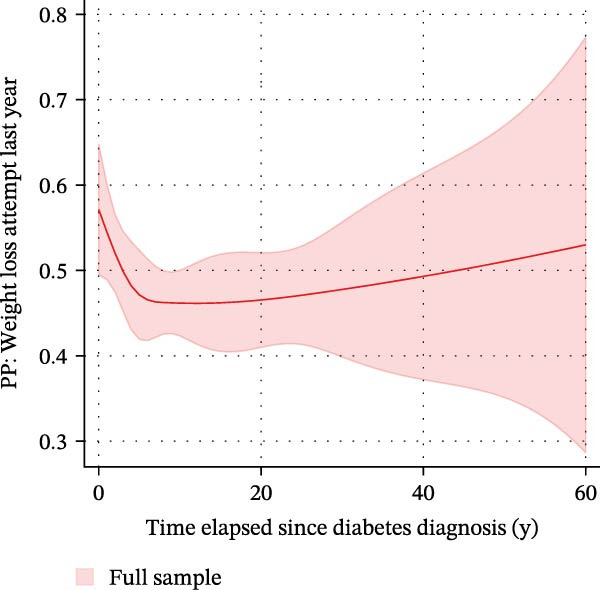
(A)

**Figure:**
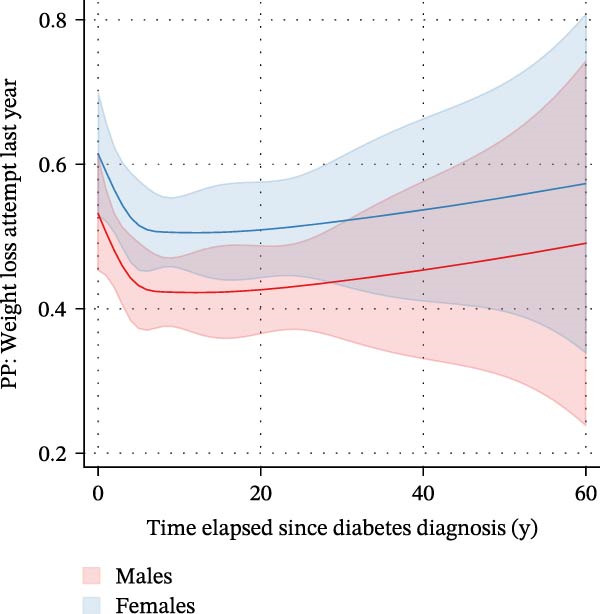
(B)

**Figure:**
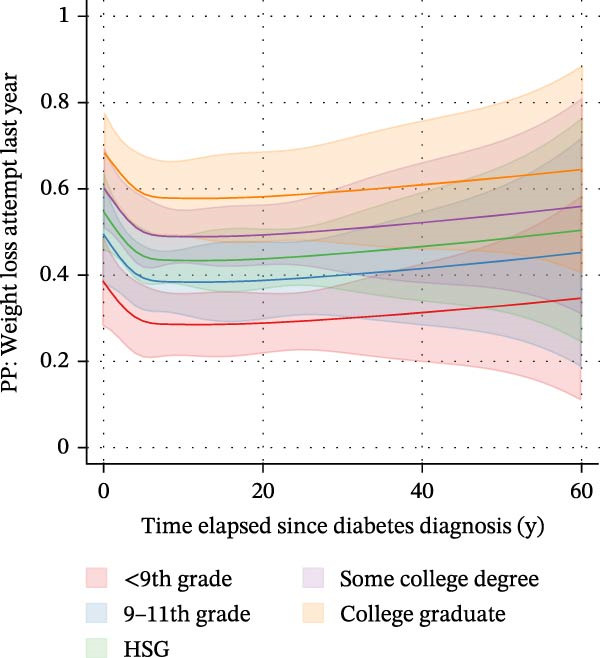
(C)

**Figure:**
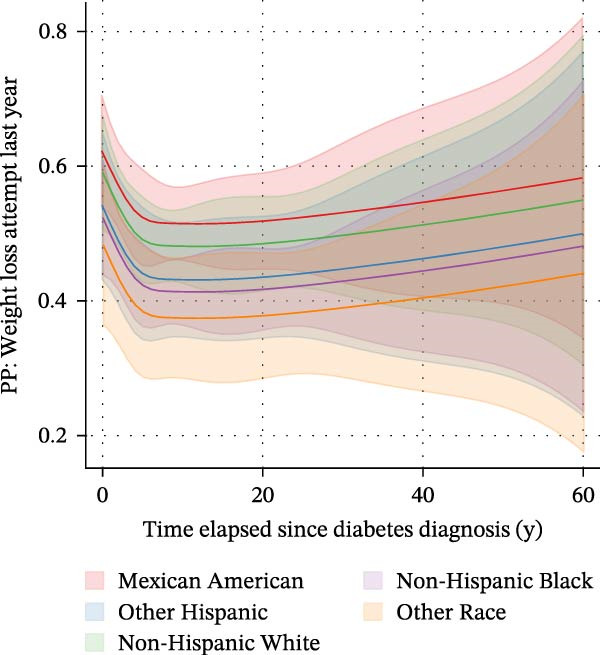
(D)

We finally used negative binomial regression to estimate the number of WLAs taken within the last 12 months depending on the time elapsed since diagnosis in those *n* = 871 participants with type 2 diabetes who attempted to lose weight (Figure 5). Both a crude and an adjusted model suggested that time elapsed since diagnosis was an important predictor (*β*‐coefficients −0.007 (CI: −0.013–(−0.002), *p* = 0.013) and −0.007 (CI: −0.013–(−0.001), *p* = 0.022), respectively). Both models suggested that the number of WLAs taken within the last year decreased with a longer time elapsed since diabetes diagnosis. When examining associations between the time elapsed since diagnosis and *specific* WLAs, we found an association with drinking a lot of water to lose weight (Supporting Information 1: Figure S5). In a logistic regression model with restricted cubic splines with *n* = 5 knots, time elapsed since diabetes diagnosis showed a nonlinear relationship with drinking high amounts of water to lose weight. Marginal predicted probabilities were highest in the first year after diagnosis: 0.25 (0.16–0.35) for males and 0.37 (0.25–0.48) for females; they were significantly lower 5 years after diagnosis: 0.11 (0.07–0.14) and 0.17 (0.11–0.23). This difference was significant in both sexes (*p* = 0.006 and 0.003, respectively).

Figure 5Marginal predicted probabilities for the number of weight loss actions taken within the last 12 months depending on the time elapsed since diagnosis in *n* = 871 participants with type 2 diabetes in the NHANES (2009–2018). (A) depicts the full sample of *n* = 871 participants with type 2 diabetes undertaking weight loss action and is based on a crude negative binomial regression model. (B) depicts the number of actions taken within the last 12 months by sex depending on the time elapsed since diagnosis after adjustment for poverty status, educational level, and marital status. In both models, time elapsed since diagnosis was an important predictor [(A) *β*‐coefficient: −0.007 (CI: −0.013 to −0.002), *p* = 0.013; (B) coefficient: −0.007 (CI: −0.013 to −0.001], *p* = 0.022), and the number of actions taken in the last year decreased with a longer time elapsed since diabetes diagnosis.

**Figure:**
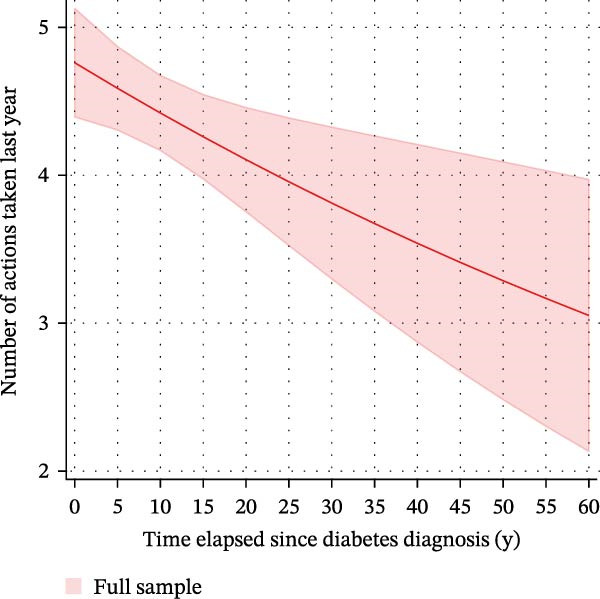
(A)

**Figure:**
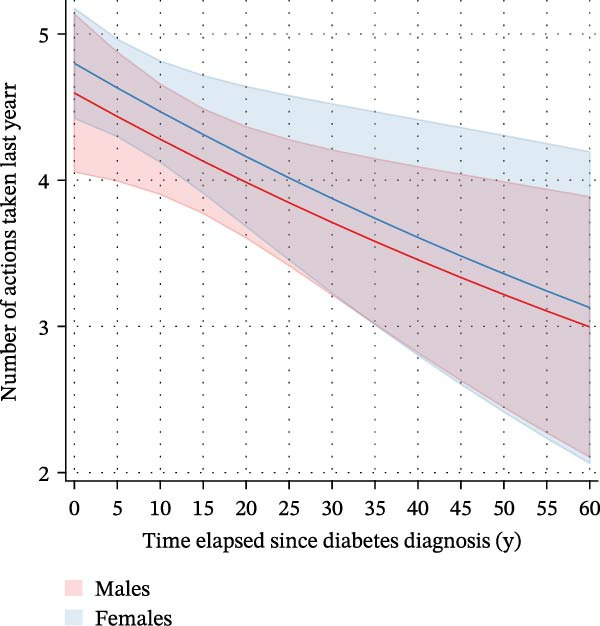
(B)

## 4. Discussion

Being diagnosed with type 2 diabetes represents a potential “teachable moment,” which may trigger positive health behavior changes in affected individuals [14]. The present study examined whether the time elapsed since a type 2 diabetes diagnosis was associated with WLAs undertaken by participants with type 2 diabetes in the NHANES (2009–2018). We further explored potential associations with the number of WLAs taken and investigated which weight loss measures were particularly popular among NHANES participants with type 2 diabetes.

Results suggested a nonlinear relationship between diabetes duration and the marginal predicted probability for undertaking WLAs in patients with type 2 diabetes. A longer time elapsed since diagnosis was associated with a lower marginal predicted number of actions taken within the last 12 months. Eating less to lose weight and exercising more were the most frequently reported weight loss strategies reported by individuals with type 2 diabetes who tried to lose weight, with 68.78% and 45.73%, respectively. Harmful practices, such as taking laxatives/vomiting or smoking, were reported by 1.31% and 1.17%, respectively.

Our findings align with several previous studies, which were also based on earlier NHANES data. Wang et al. [10] reported a weight loss intention in 60% of participants with type 2 diabetes in earlier NHANES cycles (2005–2012). While the herein reported weighted proportion of participants indicating WLAs in the past year was slightly lower (48.25%), Wang et al. used different inclusion criteria [10], which may have contributed to this difference. Potentially harmful practices, such as taking laxatives/vomiting, were equally prevalent in the Wang study. As for the WLAs taken, Mokari‐Yamchi and Rosenkranz [11] reported that exercising and eating less to lose weight were the most popular actions in the US general population who tried to lose weight, with 60.6% and 60.4%, respectively. According to our analysis, eating less to lose weight was much more frequently reported (68.78%) than exercising (45.73%). These two lifestyle modifications also emerged as the primary methods to lose weight in the previous study of Wang et al. [10] in individuals with type 2 diabetes. Such information is generally of high importance for the design of population‐based weight loss and health promotion strategies, as areas for optimizing lifestyle practices are identified. This implies both areas that are apparently popular and well‐taken (changes in diet and physical activity) as well as actions that are harmful (e.g., the use of laxatives and vomiting). Public health strategies could build on these foundations to give evidence‐based recommendations.

Overall, our findings differ largely from previous trials with regard to the effect of diabetes duration on the probability of undertaking WLAs. Several studies found no association between specific WLAs and time elapsed since a diabetes diagnosis. Hackett et al. [14] used data from the English Longitudinal Study of Ageing and investigated self‐reported behavior from *n* = 6877 community‐dwelling individuals. Their analysis suggested no evidence of positive health behavior changes subsequent to a type 2 diabetes diagnosis other than a reduction in smoking [14]. An analysis by Chong et al. [29] based on data from the New South Wales 45 and Up Study and a follow‐up study supported these findings. Participants with incident type 2 diabetes reported only minimal changes in lifestyle factors after receiving their diagnosis. Again, they were more likely to quit smoking and reported a decreased sitting duration. Overall, however, no other substantial lifestyle modifications with regard to weight management were observed.

A Norwegian online‐based study by Hansen et al. suggested that lifestyle modifications decrease with a longer diabetes duration [12]. Surveying and analyzing data from *n* = 540 individuals, the authors reported that in those participants with a disease duration of less than 10 years, 56% reported lifestyle modifications. This number dropped substantially to 33.4% in those participants with a type 2 diabetes duration ≥10 years.

The most comprehensive study in this research area was probably conducted by Gaggero et al. [12]. Using a complex fuzzy regression discontinuity design, the authors investigated lifestyle behaviors in Spanish patients with type 2 diabetes. The authors reported a short‐term reduction in body weight as a response to the type 2 diabetes diagnosis [12]. On average, participants lost 1 BMI point shortly after receiving the diagnosis. Notably, the authors also reported long‐term effects up to 3 years after the diagnosis, potentially driven by an increase in exercising and a lower carbohydrate intake. Notably, this study provided no insights into the precise WLAs taken.

Our results also raise a potential role for the time elapsed since diagnosis as a crucial factor (Figure 4). When estimating marginal predicted probabilities, we found that the probability of WLAs was highest in the first years after diagnosis, e.g., 0.57 (95% CI: 0.50–0.65) in the first 12 months. Probabilities fell thereafter, reached 0.46 after 7 years (95% CI: 0.42–0.50) and the nadir after 12 years (0.46; 95% CI: 0.41–0.51). Most notably, the time frame from 3 years to 6 years after diagnosis was identified as a potentially crucial intervention window, as marginal predicted probabilities dropped by 0.02 (95% CI: −0.38–(−0.001)) for each additional year after diagnosis within that time (*p* = 0.042).

This suggests that population‐based weight loss strategies should particularly target this time window, before susceptibility and self‐reported weight loss willingness/attempts decrease further. Sustainable and long‐lasting lifestyle modifications require time and perseverance. Against this background, it appears reasonable to tailor lifestyle‐centered public health strategies that take a potential decline in motivation into account and that target individuals with type 2 diabetes *before* they lose motivation to lose weight [16–18].

Weight loss is of paramount importance in type 2 diabetes and essential to improve glycemic control. While effective pharmacological approaches exist these days, substantial public health efforts and strategies to combat the obesity and diabetes epidemic are warranted to decrease healthcare costs [9]. According to a recent meta‐analysis by Kanbour et al. [30], a robust dose–response relationship between bodyweight loss and diabetes remission exists—independent of factors such as age, HbA1c, BMI, and type of intervention. Striving for early weight loss is thus probably *the* most important clinical goal in patients with type 2 diabetes to combat mortality and comorbidities from early on [31, 32]. Adopting lifestyle changes is of paramount importance to reach this particular goal. Our study may thus provide guidance for policy makers, health professionals, health administrations, and public health practitioners to achieve that goal by suggesting an evidence‐based optimal intervention window to maximize the impact of population‐based weight loss measures in patients with type 2 diabetes.

The limitations of this analysis include the cross‐sectional design of the study (which does not allow for causal inferences) and the fact that WLAs were only queried within the last 12 months. Most data were self‐reported, which may have introduced recall and reporting bias. This applies in particular to the time elapsed since diabetes diagnosis, which was not drawn from medical records. While numerous sociodemographic factors were adjusted for, we transparently acknowledge that some potentially unmeasured confounders remain. These include certain comorbidities (e.g., cardiovascular disease and healthcare access). While the data dates back to 2011, when GLP‐1 receptor agonists were not as widely used as today, this is another limitation worth noting. Finally, we did not adjust for the BMI in the respective multivariate logistic regression models because BMI was deemed to be a relevant mediator instead of a confounder.

As for the strengths, we used data from a nationally representative survey with a modest sample size, which may be extrapolated to represent *n* = 12,825,867 US Americans. The regression model with restricted cubic splines allowed us to model nonlinear relationships and, in combination with Stata’s margins function, to make predictions about an optimal intervention window for population‐based weight loss measures in patients with type 2 diabetes. Nevertheless, additional prospective studies are necessary for better insights into lifestyle changes in patients with type 2 diabetes and their impact on weight and glycemic control.

## 5. Conclusions

Weight loss in patients with type 2 diabetes is of utmost importance to improve glycemic control and to ideally induce diabetes remission. The present study identified the importance of the time elapsed since diabetes diagnosis as an important parameter that must be taken into account for population‐based weight loss strategies in patients with type 2 diabetes. Time elapsed since diagnosis showed a nonlinear relationship with the odds for undertaking weight loss incentives, which may have important clinical and public health implications. The time frame from 3 years to 6 years after diagnosis was identified as a potentially crucial time window, as marginal predicted probabilities for weight loss attempts dropped by 0.02 (95% CI: −0.38–(−0.001)) for each additional year after diagnosis within that time (*p* = 0.042).

**Nomenclature**
NCHS: National Center for Health Statistics
NHANES: National Health and Nutrition Examination Surveys
WLAs: Weight Loss Actions.

## Author Contributions

Conceptualization, data curation, formal analysis, funding acquisition, investigation, methodology, software, visualization, writing – original draft: Maximilian Andreas Storz. Project administration, validation, writing – review and editing: Maximilian Andreas Storz, Roman Huber, and Jochen Seufert. Resources: Maximilian Andreas Storz and Roman Huber. Supervision: Roman Huber and Jochen Seufert.

## Acknowledgments

No LLM/AI tools were used for this submission.

## Funding

The authors received no financial support/funding. Open Access funding enabled and organized by Projekt DEAL.

## Disclosure

This work has not been published before; it is not under consideration for publication anywhere else. This work has been approved by all coauthors. All authors reviewed and approved the final manuscript. All authors agreed to be accountable for all aspects of the work in ensuring that questions related to the accuracy or integrity of any part of the work are appropriately investigated and resolved.

## Ethics Statement

NHANES was approved by the National Centre for Health Statistics Research Ethics Review Board.

## Consent

Written informed consent was obtained from all NHANES participants (https://www.cdc.gov/nchs/nhanes/about/erb.html).

## Conflicts of Interest

The authors declare no conflicts of interest.

## Supporting Information

Additional supporting information can be found online in the Supporting Information section.

## Supporting information


**Supporting Information 1** Table S1: NHANES variables used in this study. Figure S1: Histograms depicting the distribution of age at diabetes diagnosis (panel A) and the time elapsed since diabetes diagnosis (panel B) in the full sample comprising *n* = 2118 participants with type 2 diabetes in the NHANES (2009−2018). Figure S2: Histogram displaying the distribution of the number of weight loss actions taken among *n* = 871 participants with type 2 diabetes and a weight loss attempt in the past 12 months in the NHANES (2009−2018). Figure S3: Histograms displaying the distribution of body weight differences at the NHANES examination vs. 12 months ago. Panel A shows the difference between self‐reported body weight at participation vs. self‐reported weight 12 months ago. Negative numbers indicate weight loss; positive numbers indicate weight gain. Panel B shows the difference between measured body weight at participation vs. self‐reported weight 12 months ago. Figure S4: Marginal predicted probabilities for an attempt to lose weight within the last 12 months depending on the time elapsed since diabetes diagnosis. Figure S5: Marginal predicted probabilities for drinking a lot of water to lose weight within the last 12 months depending on the time elapsed since diabetes diagnosis based on a logistic regression model with restricted cubic splines with 5 knots.

**Supporting Information 2** Table S2: STROBE checklist.

## Data Availability

Data are publicly available online (https://wwwn.cdc.gov/nchs/nhanes/Default.aspx). The datasets used and analyzed during the current study are available from the corresponding author (MAS) upon request.
