# Supplementary material for: Weight Loss Strategies Associated With Type 2 Diabetes Duration: A Population‐Based Optimal Intervention Window From the NHANES
Source: J Diabetes Res. 2026 Apr 27;2026:8319702. doi: 10.1155/jdr/8319702 (PMC13121560; doi:10.1155/jdr/8319702)

# Supplementary Tables

## Supplementary Table 1

Supplementary Table 1 title: NHANES variables used in this study

| NHANES variable | Type of variable |
| --- | --- |
| RIAGENDR - Gender | Categorical variable: 1 = Male; 2 = Female |
| RIDAGEYR - Age at Screening | Continuous variable |
| RIDRETH1 - Race/Ethnicity | Categorical variable: 1 = Mexican American; 2 = Other Hispanic; 3 = Non-Hispanic Black; 4 = Non-Hispanic White; 5 = Other Race |
| DMDEDUC2 - Education Level - Adults 20+ | Categorical variable: 1 = Less Than 9th Grade; 2 = 9-11th Grade (Includes 12th grade with no diploma); 3 = High School Grad/GED or Equivalent; 4 = Some College or AA degree; 5 = College Graduate or above |
| DMDMARTL - Marital Status | Categorical variable: 1 = Married / Living with a partner; 2 = Widowed/divorced/separated; 3 = Never married |
| DMDHHSIZ - Total number of people in the household | Continuous variable |
| INDFMPIR - Ratio of family income to poverty | Continuous variable |
| BMXWT - Weight (kg) | Continuous variable |
| BMXHT - Standing Height (cm) | Continuous variable |
| DIQ010 - Doctor told you have diabetes | Categorical variable: 1 = Yes; 2 = No; 3 = Borderline |
| DID040 - Age when first told you had diabetes | Continuous variable |
| DIQ050 - Taking insulin now | Categorical variable: 1 = Yes; 2 = No |
| WHQ030 - How do you consider your weight | Categorical variable: 1 = Overweight; 2 = Underweight; 3 = About the right weight |
| WHQ070 - Tried to lose weight in past year | Categorical variable: 1 = Yes; 2 = No |
| WHD020 - Current self-reported weight (pounds) | Continuous variable |
| WHD050 - Self-reported weight - 1 yr ago (pounds) | Continuous variable |
| WHD080A - Ate less to lose weight | Skip variable with affirmative response |
| WHD080B - Switched to foods with lower calories | Skip variable with affirmative response |
| WHD080C - Ate less fat to lose weight | Skip variable with affirmative response |
| WHD080D - Exercised to lose weight | Skip variable with affirmative response |
| WHD080E - Skipped meals | Skip variable with affirmative response |
| WHD080F - Ate diet foods or products | Skip variable with affirmative response |
| WHD080G - Used a liquid diet formula | Skip variable with affirmative response |
| WHD080H - Joined a weight loss program | Skip variable with affirmative response |
| WHD080I - Took prescription diet pills | Skip variable with affirmative response |
| WHD080J - Took non-RX suppl. to lose weight | Skip variable with affirmative response |
| WHD080K - Took laxatives or vomited | Skip variable with affirmative response |
| WHD080M - Drank a lot of water | Skip variable with affirmative response |
| WHD080N - Followed a special diet | Skip variable with affirmative response |
| WHD080O - Ate fewer carbohydrates | Skip variable with affirmative response |
| WHD080P - Started to smoke or began to smoke again | Skip variable with affirmative response |
| WHD080Q - Ate more fruits, vegetables, salads | Skip variable with affirmative response |
| WHD080R - Changed eating habits | Skip variable with affirmative response |
| WHD080S - Ate less sugar, candy, sweets | Skip variable with affirmative response |
| WHD080T - Ate less junk food or fast food | Skip variable with affirmative response |
| WHD080U - Had weight loss surgery to lose weight | Skip variable with affirmative response |
| WHD080L - Other | Skip variable with affirmative response |

## Supplementary Table 2

Supplementary Table 2 title: STROBE checklist – *uploaded separately*

# Supplementary Figures

## Supplementary Figure 1

Supplementary Figure 1 title: Histograms depicting the distribution of age at diabetes diagnosis (panel A) and the time elapsed since diabetes diagnosis (panel B) in the full sample comprising *n* = 2,118 participants with type-2-diabetes in the NHANES (2009-2018)


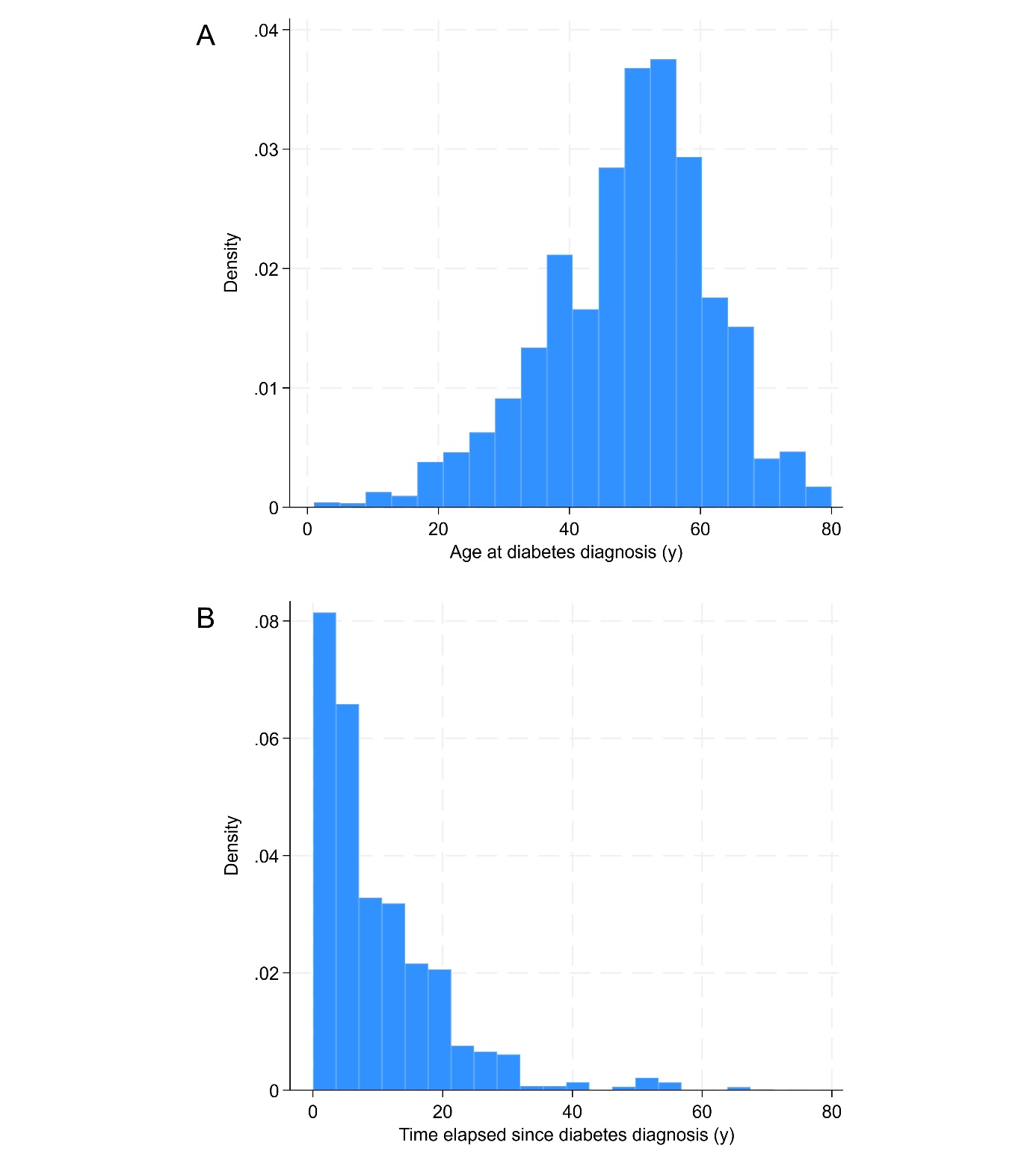


## Supplementary Figure 2

Supplementary Figure 2 title: Histogram displaying the distribution of the number of weight loss actions taken among *n* = 871 participants with type-2-diabetes and a weight loss attempt in the past 12 months in the NHANES (2009-2018)


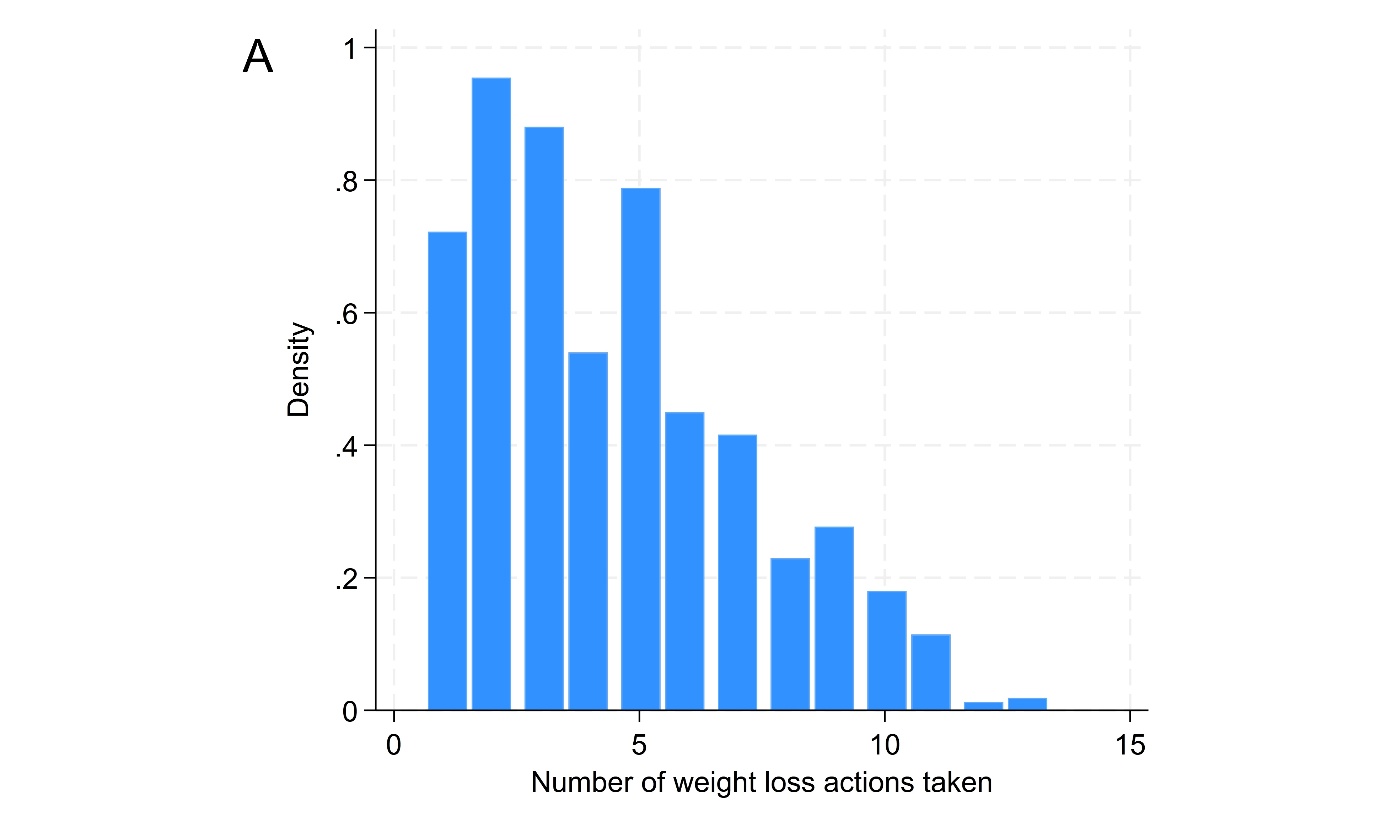


## Supplementary Figure 3

Supplementary Figure 3 title: Histograms displaying the distribution of body weight differences at the NHANES examination vs. 12 months ago. Panel A shows the difference between self-reported body weight at participation vs. self-reported weight 12 months ago. Negative numbers indicate weight loss; positive numbers indicate weight gain. Panel B shows the difference between measured body weight at participation *vs*. self-reported weight 12 months ago.


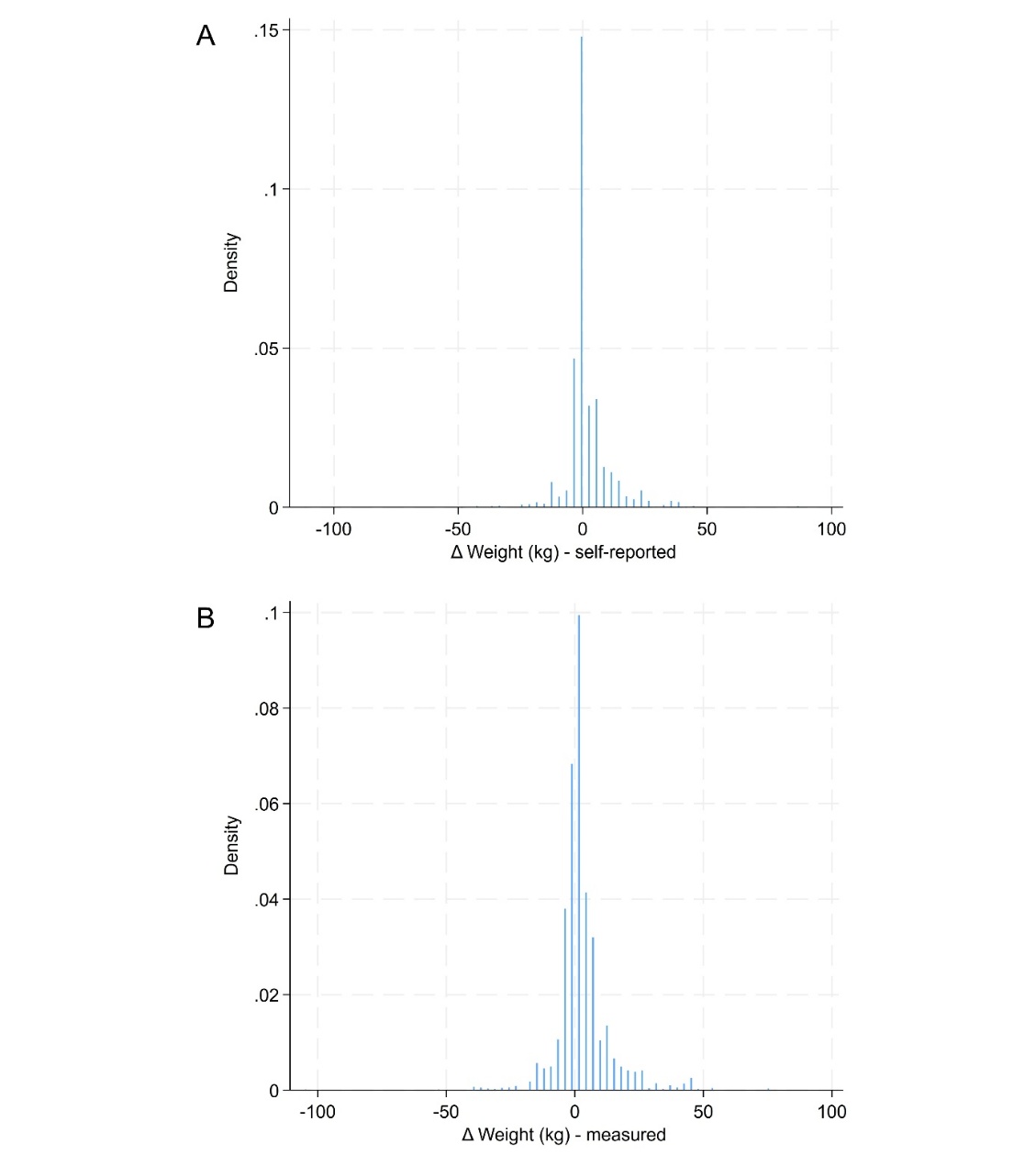


## Supplementary Figure 4

Supplementary Figure 4 title: Marginal predicted probabilities for an attempt to lose weight within the last 12 months depending on the time elapsed since diabetes diagnosis. All margins plots are based on a logistic regression model with time elapsed since diagnosis as a continuous predictor and with race/ethnicity, sex, poverty status, educational level and marital status as covariates. No cubic splines were used. Time elapsed since diabetes diagnosis was not associated with a decreased likelihood of attempting to lose weight (OR: 0.99, CI: 0.98-1.01, *p* = 0.357). Female sex (panel A) was associated with a higher likelihood of weight loss attempts within the last year (OR: 1.42, CI: 1.09-1.85, *p* = 0.011). Having a college degree or higher was also associated with a higher likelihood of weight loss attempts within the last year (OR: 1.78, CI: 1.14-2.77, *p* = 0.012) whereas not having completed the 9^th^ grade was associated with a lower likelihood (OR: 0.52, CI: 0.36-0.77, *p* = 0.001). Non-Hispanic Black ethnicity was also associated with a lower likelihood (OR: 0.76, CI: 0.58-0.99, *p* = 0.040). HSG = High School Graduate. CD = College Degree. CG = College Graduate.


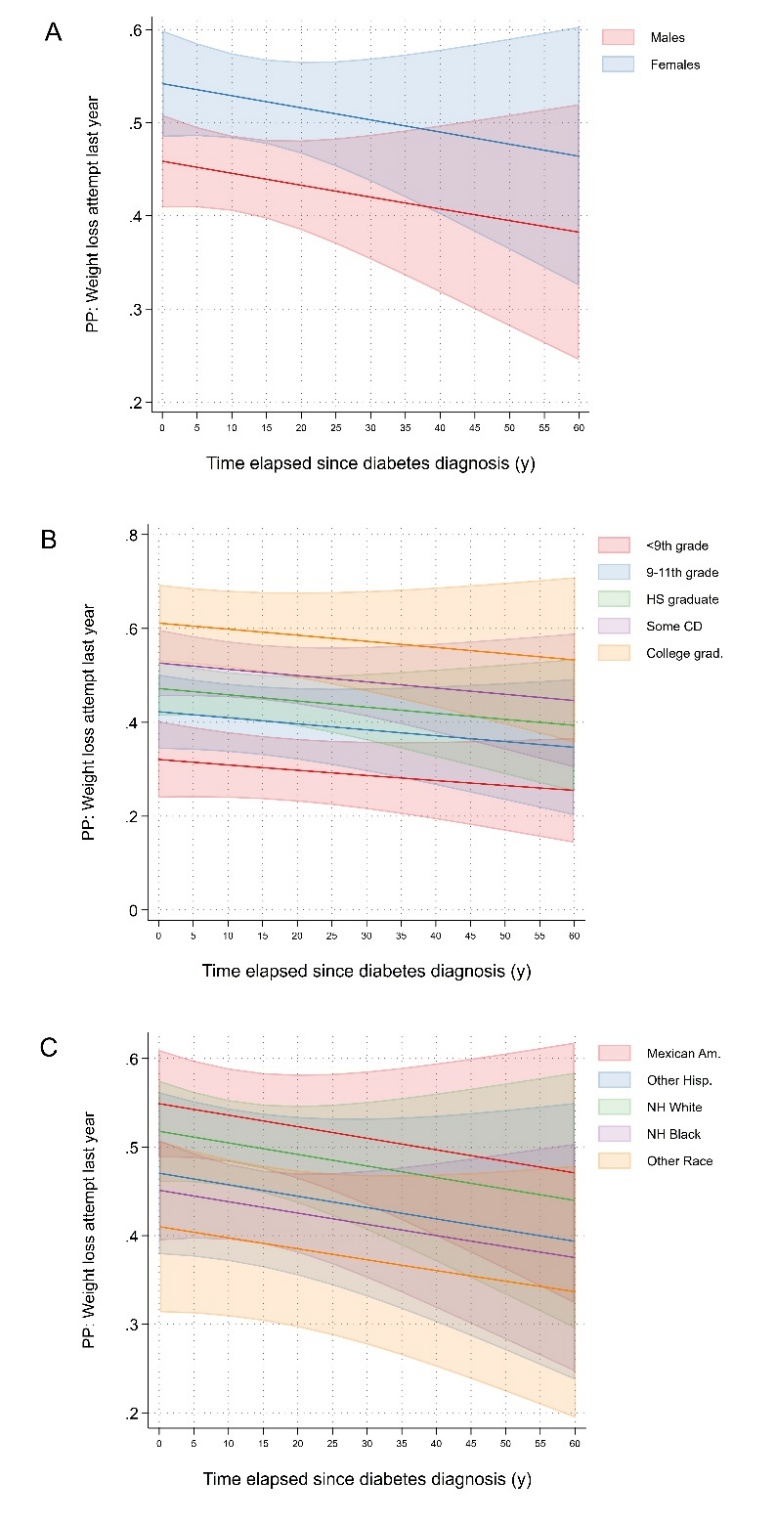


## Supplementary Figure 5

Supplementary Figure 5 title: Marginal predicted probabilities for drinking a lot of water to lose weight within the last 12 months depending on the time elapsed since diabetes diagnosis based on a logistic regression model with restricted cubic splines with 5 knots. Based on the full sample comprising n = 2,118 observations. Time elapsed since diabetes diagnosis showed and drinking high amounts of water to lose weight showed a non-linear relationship. Panel A depicts marginal predicted probabilities depending on sex, whereas panel B depicts marginal predicted probabilities depending on educational level. Marginal predicted probabilities were highest in the first year after diagnosis (0.25 (0.16 - 0.35) for males and 0.37 (0.25 - 0.48) for females and were significantly lower 5 years after diagnosis (0.11 (0.07 – 0.14) and 0.17 (0.11 - 0.23). This difference was significant (*p* = 0.006 and 0.003, respectively). HSG = High School Graduate. CD = College Degree. CG = College Graduate.


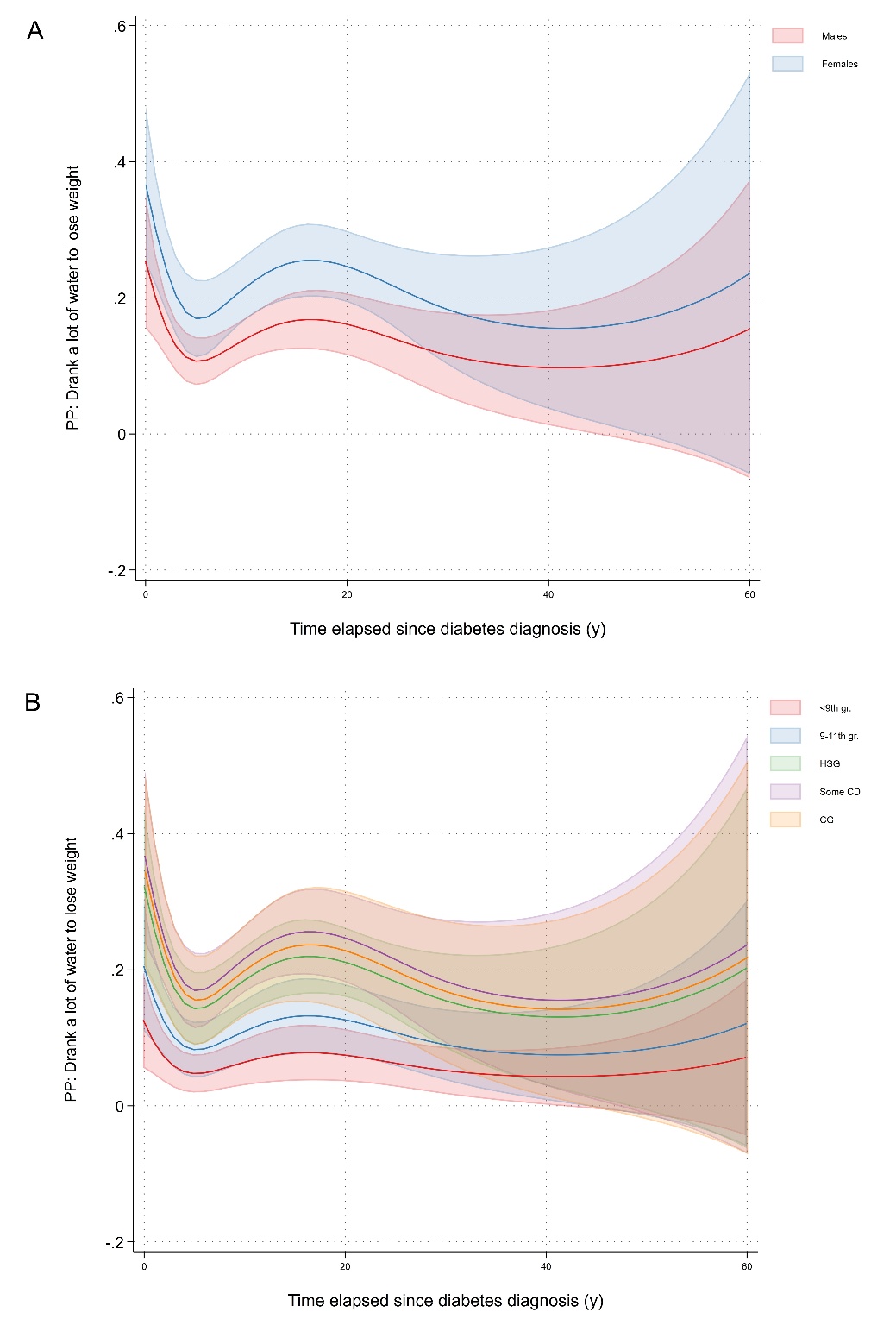

Supplement: Supplementary file 1 — Supporting Information 1 Table S1: NHANES variables used in this study. Figure S1: Histograms depicting the distribution of age at diabetes diagnosis (panel A) and the time elapsed since diabetes diagnosis (panel B) in the full sample comprising n = 2118 participants with type 2 diabetes in the NHANES (2009−2018). Figure S2: Histogram displaying the distribution of the number of weight loss actions taken among n = 871 participants with type 2 diabetes and a weight loss attempt in the past 12 months in the NHANES (2009−2018). Figure S3: Histograms displaying the distribution of body weight differences at the NHANES examination vs. 12 months ago. Panel A shows the difference between self‐reported body weight at participation vs. self‐reported weight 12 months ago. Negative numbers indicate weight loss; positive numbers indicate weight gain. Panel B shows the difference between measured body weight at participation vs. self‐reported weight 12 months ago. Figure S4: Marginal predicted probabilities for an attempt to lose weight within the last 12 months depending on the time elapsed since diabetes diagnosis. Figure S5: Marginal predicted probabilities for drinking a lot of water to lose weight within the last 12 months depending on the time elapsed since diabetes diagnosis based on a logistic regression model with restricted cubic splines with 5 knots. [file JDR-2026-8319702-s001.docx]
