# Supplementary material for: Weight Loss Strategies Associated With Type 2 Diabetes Duration: A Population‐Based Optimal Intervention Window From the NHANES
Source: J Diabetes Res. 2026 Apr 27;2026:8319702. doi: 10.1155/jdr/8319702 (PMC13121560; doi:10.1155/jdr/8319702)
Supplement: Supplementary file 2 — Supporting Information 2 Table S2: STROBE checklist. [file JDR-2026-8319702-s002.doc]

STROBE Statement—checklist of items that should be included in reports of observational studies

|  | Item No | Recommendation |
| --- | --- | --- |
| **Title and abstract** | 1 | (*a*) Indicate the study’s design with a commonly used term in the title or the abstract  *-> The methods section of the abstract specifies that cross-sectional data from the NHANES were used.* |
|  |  | (*b*) Provide in the abstract an informative and balanced summary of what was done and what was found  *-> see page 2, abstract. Multivariate logistic regression models with restricted cubic splines were used to estimate marginal predicted probabilities for undertaking weight loss action in n=2,118 NHANES participants with type-2-diabetes depending on the time elapsed since diagnosis.* |
| Introduction | | |
| Background/rationale | 2 | Explain the scientific background and rationale for the investigation being reported  *-> see page 3, paragraphs 1 – 4 of the introduction section. The rationale for this analysis was the lack of studies (and the conflicting results of the few available studies) investigating potential associations between the time elapsed since a type-2-diabetes diagnosis and the odds for undertaking weight loss actions.* |
| Objectives | 3 | State specific objectives, including any prespecified hypotheses  *-> page 3, last paragraph. “More specifically, we hypothesized that the odds for undertaking weight loss actions would decrease with a longer time elapsed since type-2-diabetes diagnosis (primary hypothesis). We also hypothesized that the time elapsed since diagnosis would be associated with a lower number of weight loss actions taken. Finally, we conducted an exploratory analysis to identify the most frequent weight loss actions reported by NHANES participants with type-2-diabetes, and investigated whether the predicted probabilities for certain (potentially harmful) actions increase with a longer time elapsed since diagnosis”.* |
| Methods | | |
| Study design | 4 | Present key elements of study design early in the paper  *-> key elements were described early in the paragraph NHANES 2008-2019 in the methods section on page 4* |
| Setting | 5 | Describe the setting, locations, and relevant dates, including periods of recruitment, exposure, follow-up, and data collection  *-> key elements were described early in the paragraph NHANES 2008-2019 in the methods section on page 4* |
| Participants | 6 | *Cross-sectional study*—Give the eligibility criteria, and the sources and methods of selection of participants  *-> all criteria were described in a dedicated paragraph in the methods section called “in- and exclusion criteria”. We also provided a participant inclusion flowchart.* |
|  |  | *-* |
| Variables | 7 | Clearly define all outcomes, exposures, predictors, potential confounders, and effect modifiers. Give diagnostic criteria, if applicable  *-> see sections on “primary outcome” and “secondary outcome” as well as exposure.* |
| Data sources/ measurement | 8* | For each variable of interest, give sources of data and details of methods of assessment (measurement). Describe comparability of assessment methods if there is more than one group  -> *discussed in each subsection as well as in the covariable section.* |
| Bias | 9 | Describe any efforts to address potential sources of bias |
| Study size | 10 | Explain how the study size was arrived at  *-> see Figure 1* |
| Quantitative variables | 11 | Explain how quantitative variables were handled in the analyses. If applicable, describe which groupings were chosen and why  *-> continuous variables were not grouped/categorized.* |
| Statistical methods | 12 | (*a*) Describe all statistical methods, including those used to control for confounding  *-> page 5, section on “statistical methods”* |
|  |  | (*b*) Describe any methods used to examine subgroups and interactions  *-> see above. No interactions were used. Subgroups were examined using unconditional subclass analysis.* |
|  |  | (*c*) Explain how missing data were addressed  *-> participants with missing data were excluded.* |
|  |  | *Cross-sectional study*—If applicable, describe analytical methods taking account of sampling strategy  *-> see paragraph on NHANES sampling* |
|  |  | (*e*) Describe any sensitivity analyses  *-> not performed.* |

| Results | | |
| --- | --- | --- |
| Participants | 13* | (a) Report numbers of individuals at each stage of study—eg numbers potentially eligible, examined for eligibility, confirmed eligible, included in the study, completing follow-up, and analysed  *-> see Figure 1 and Table 1. See paragraph 1 of the results section.* |
|  |  | (b) Give reasons for non-participation at each stage *-> see Figure 1* |
|  |  | (c) Consider use of a flow diagram *-> see Figure 1* |
| Descriptive data | 14* | (a) Give characteristics of study participants (eg demographic, clinical, social) and information on exposures and potential confounders  *-> see Table 1* |
|  |  | (b) Indicate number of participants with missing data for each variable of interest  *-> not applicable* |
|  |  | (c) *Cohort study*—Summarise follow-up time (eg, average and total amount) *-> not applicable* |
| Outcome data | 15* | *Cross-sectional study—*Report numbers of outcome events or summary measures |
|  |  | *-> see Table 1* |
|  |  |  |
| Main results | 16 | (*a*) Give unadjusted estimates and, if applicable, confounder-adjusted estimates and their precision (eg, 95% confidence interval). Make clear which confounders were adjusted for and why they were included *-> provided in all analyses. OR and 95% were reported throughout. Confounders were clearly specified in the methods section and in the legend of the respective figure.* |
|  |  | (*b*) Report category boundaries when continuous variables were categorized *-> not applicable* |
|  |  | (*c*) If relevant, consider translating estimates of relative risk into absolute risk for a meaningful time period *-> not applicable* |
| Other analyses | 17 | Report other analyses done—eg analyses of subgroups and interactions, and sensitivity analyses *-> see popular weight loss actions in the subgroup of participants with weight loss intentions and type-2-diabetes. See analyses using restricted cubic splines.* |
| Discussion | | |
| Key results | 18 | Summarise key results with reference to study objectives  *-> Page 6, first paragraph of the discussion section.* |
| Limitations | 19 | Discuss limitations of the study, taking into account sources of potential bias or imprecision. Discuss both direction and magnitude of any potential bias  *-> “The present study is not without limitations. These include the cross-sectional design of the study (which does not allow for causal inferences) and the fact that weight loss actions were only queried within the last 12 months. Most data was self-reported, which may have introduced recall and reporting bias. As for the strengths, we used data from a nationally representative survey with a modest sample size which may be extrapolated to represent n = 12,825,867 US Americans. The regression model with restricted cubic splines allowed us to model non-linear relationships, and, in combination with Stata’s margins function, to make predictions about an optimal intervention window for population-based weight loss measures in patients with type-2-diabetes. Nevertheless, additional prospective studies are necessary for better insights into lifestyle changes in patients with type-2-diabetes and their impact on weight and glycemic control.”* |
| Interpretation | 20 | Give a cautious overall interpretation of results considering objectives, limitations, multiplicity of analyses, results from similar studies, and other relevant evidence  *-> “Weight loss in patients with type-2-diabetes is of utmost importance to improve glycemic control and to ideally induce diabetes remission. The present study emphasized the importance of the time elapsed since diabetes diagnosis as an important parameter that must be taken into account for population-based weight loss strategies in patients with type-2-diabetes. Time elapsed since diagnosis showed a non-linear relationship with the odds for undertaking weight loss incentives, which may have important clinical and public health implications. The time frame from three years to six years after diagnosis was identified as a potentially crucial time window, as marginal predicted probabilities for weight loss attempts dropped by 0.02 (95%-CI: -0.38 – (-0.001)) for each additional year after diagnosis within that time (p = 0.042).”* |
| Generalisability | 21 | Discuss the generalisability (external validity) of the study results  *-> NHANES data allows for nationally representative assessments.* |
| Other information | | |
| Funding | 22 | Give the source of funding and the role of the funders for the present study and, if applicable, for the original study on which the present article is based *-> no funding received* |

*Give information separately for cases and controls in case-control studies and, if applicable, for exposed and unexposed groups in cohort and cross-sectional studies.

**Note:** An Explanation and Elaboration article discusses each checklist item and gives methodological background and published examples of transparent reporting. The STROBE checklist is best used in conjunction with this article (freely available on the Web sites of PLoS Medicine at http://www.plosmedicine.org/, Annals of Internal Medicine at http://www.annals.org/, and Epidemiology at http://www.epidem.com/). Information on the STROBE Initiative is available at www.strobe-statement.org.
